# Supplementary material for: A new fMRI quality metric using multi-echo information: Theory, validation and implications
Source: bioRxiv. 2026 Mar 23:2026.03.19.712948. Preprint. [Version 1] doi: 10.64898/2026.03.19.712948 (PMC13041840; doi:10.64898/2026.03.19.712948)
Supplement: Supplement 1 [file NIHPP2026.03.19.712948v1-supplement-1.pdf]

bioRxiv preprint doi: https://doi.org/10.64898/2026.03.19.712948; this version posted March 23, 2026. The copyright holder for this preprint (which was not certified by peer review) is the author/funder. This article is a US Government work. It is not subject to copyright under 17 USC 105 and is also made available for use under a CC0 license.

Supplementary Figures

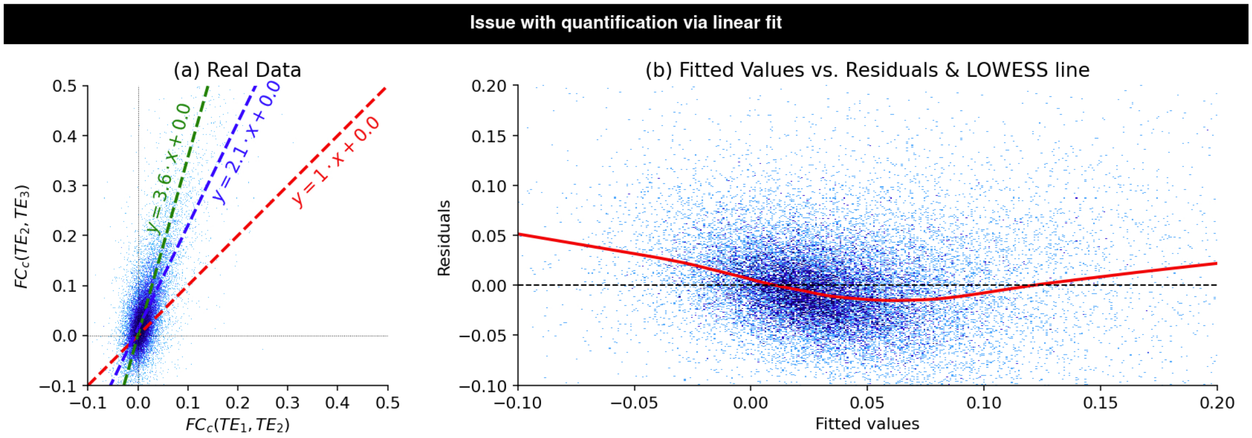

Supplementary Figure 1. Representative dataset showing why a simple linear fit to the data is not a robust way to quantify where a given FC_C matrix sits relative to the two signal regimes of interest. (a) Scatter plot of FC_C for two separate pairs of echo times. Edges are depicted as blue dots. Green dashed line shows expected behavior for BOLD dominated data. Red dashed line (identity line) shows expected behavior for non-BOLD dominated data. Blue dashed line shows the linear fit to the point cloud associated with input FC_C. Although the data primarily sits over the green dashed line, the linear fit might suggest differently. (b) Scatter plot of residual values for the linear fit as a function of fitted values. We can observe that residuals are not homogenously distributed, which signals heteroscedasticity. The red line corresponds to the locally weighted scatter plot smoothing (LOWESS) fit to the cloud point.

bioRxiv preprint doi: https://doi.org/10.64898/2026.03.19.712948; this version posted March 23, 2026. The copyright holder for this preprint (which was not certified by peer review) is the author/funder. This article is a US Government work. It is not subject to copyright under 17 USC 105 and is also made available for use under a CC0 license.

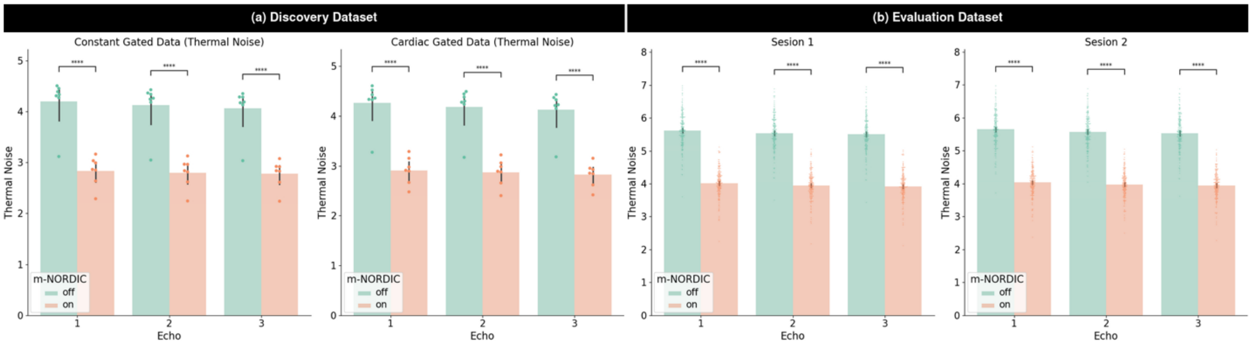

Supplementary Figure 2. Reduction in thermal noise obtained using m-NORDIC. (a) Results for the discovery dataset with constant-gated scans on the left and cardiac-gated scans on the right. (b) Results for the evaluation dataset with scans from session 1 on the left and scans from session 2 on the right. In all plots colored bars indicate average values across the sample, error bars the 95% confidence interval and dots the results for each individual scan. Statistical annotations correspond to a double-sided paired T-test (****: $p < 1e^{-4}$).

bioRxiv preprint doi: https://doi.org/10.64898/2026.03.19.712948; this version posted March 23, 2026. The copyright holder for this preprint (which was not certified by peer review) is the author/funder. This article is a US Government work. It is not subject to copyright under 17 USC 105 and is also made available for use under a CC0 license.

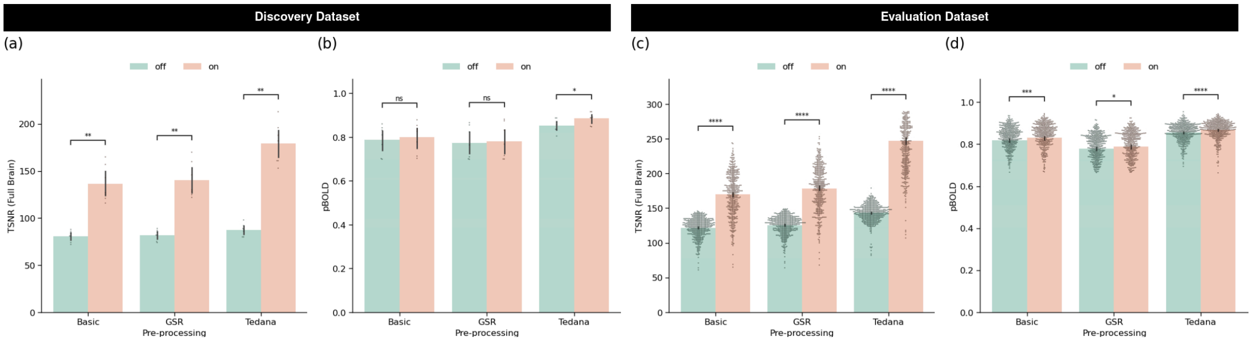

Supplementary Figure 3. Changes in TSNR and p_{BOLD} when applying m-NORDIC. (a) and (b) TSNR and p_{BOLD} results for the discovery dataset, respectively. It includes only constant-gated data. (c) and (d) TSNR and p_{BOLD} results for the evaluation dataset. It includes data from both sessions. Statistical annotations correspond to a double-sided Mann-Whitney Test (ns = not significant; * p<0.05; ** p<0.01; *** p<0.001; **** p< 1e^{-4}.

bioRxiv preprint doi: https://doi.org/10.64898/2026.03.19.712948; this version posted March 23, 2026. The copyright holder for this preprint (which was not certified by peer review) is the author/funder. This article is a US Government work. It is not subject to copyright under 17 USC 105 and is also made available for use under a CC0 license.

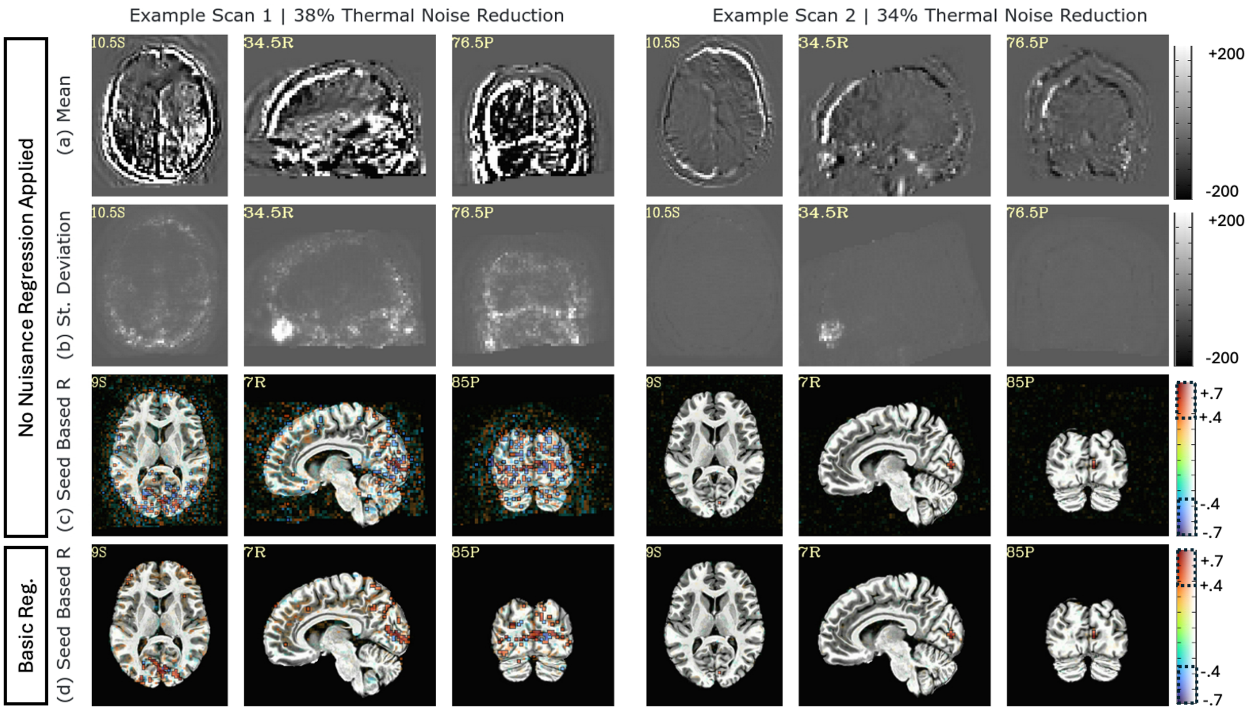

Supplementary Figure 4. Effects of m-NORDIC denoising for the two scans that had the largest amount of thermal noise removed. All maps in this figure were computed using the differential timeseries between having applied or not applied NORDIC. Results are presented following spatial normalization into MNI space (top three columns) and following Basic Nuisance regression (bottom column). In theory, if m-NORDIC only removed random thermal noise, no spatial or correlational structure should be observed. (a) Mean across time of the differential timeseries for two exemplary scans. Both maps show clear structure, although the magnitude of that structure is much higher on the right. (b) Standard deviation across time of the differential timeseries. The map on the right shows a clear spatial structure, while for the scan on the left, structure is only appreciated near the eyes. (c) Seed-based correlation for a seed voxel in visual cortex prior to any nuisance regression. Clear correlational structure is observed on the right scan, but not the left. (d) Seed-based correlation for a seed voxel in visual cortex following Basic nuisance regression. Clear correlational structure is observed on the right scan, but not the left.
